# Genome-wide identification, classification and expression analysis of the JmjC domain-containing histone demethylase gene family in *Jatropha curcas* L.

**DOI:** 10.1038/s41598-022-10584-3

**Published:** 2022-04-21

**Authors:** Jie Wang, Xiaoke Jiang, Hanrui Bai, Changning Liu

**Affiliations:** 1 CAS Key Laboratory of Tropical Plant Resources and Sustainable Use, Xishuangbanna Tropical Botanical Garden, Chinese Academy of Sciences, Kunming, 650223 China; 2 College of Life Sciences, University of Chinese Academy of Sciences, Beijing, 100049 China; 3 College of Life Sciences, Division of Life Sciences and Medicine, University of Science and Technology of China, Hefei, 230026 China; 4 Center of Economic Botany, Core Botanical Gardens, Chinese Academy of Sciences, Menglun, MenglaYunnan, 666303 China

**Keywords:** Evolution, Plant sciences

## Abstract

JmjC domain-containing proteins, an important family of histone lysine demethylase, play significant roles in maintaining the homeostasis of histone methylation. In this study, we comprehensively analyzed the JmjC domain-containing gene family in *Jatropha curcas* and found 20 JmjC domain-containing genes (JcJMJ genes). Phylogenetic analysis revealed that these JcJMJ genes can be classified into five major subgroups, and genes in each subgroup had similar motif and domain composition. Cis-regulatory element analysis showed that the number and types of cis-regulatory elements owned by the promoter of JcJMJ genes in different subgroup were significantly different. Moreover, miRNA target prediction result revealed a complicated miRNA-mediated post-transcriptional regulatory network, in which JcJMJ genes were regulated by different numbers and types of miRNAs. Further analysis of the tissue and stress expression profiles showed that many JcJMJ genes had tissue and stress expression specificity. All these results provided valuable information for understanding the evolution of JcJMJ genes and the complex transcriptional and post transcriptional regulation involved, and laid the foundation for further functional analysis of JcJMJ genes.

## Introduction

Histones are subject to a wide variety of post-translational modifications, including phosphorylation, ubiquitination, citrullination, SUMO modification, ADP ribosylation, methylation, and acetylation^1–4^. Among them, histone methylation and demethylation, often referred to as the "second genetic code", play important roles in regulating transcription, genome integrity and epigenetics^5–7^. Histone methylation can occur on a variety of lysine and arginine residues and is primarily catalyzed by a family of proteins containing PRMT and SET domains^8–10^. Histone demethylation involves two types of demethylase. The first one is Lysine-specific demethylase 1 (LSD1), which is a member of the flavin-dependent amine oxidase family of enzymes. The second family of histone demethylases has a JmjC domain, which catalyzes histone lysine demethylation through the oxidation of ferrous ions (Fe (II)) and α- ketoglutarate (α-kg)^7,11,12^.

JmjC domain-containing protein was first discovered in a mouse mutant with a "cross-shaped" neural plate, and has been reported in humans, yeast and plants since then. As a class of important histone demethylases in plants and animals, JmjC domain-containing protein plays important roles in histone modifications^6,13,14^. JmjC domain-containing proteins have been classified into eight groups in animals and five groups in plants^11^. In Arabidopsis, JmjC domain proteins could be divided into KDM4/JHDM3 group, KDM5/JARID1 group, JHDM6/JMJD6 group, KDM3/JHDM2 group, and JmjC domain-only group^15^. Among these different groups, members of the JmjC domain-only group only contain JmjC domains. While members in other groups contain not only the JmjC domain but also other domains such as JmjN, ARID, FYRN, FYRC, zf-C5HC2, F-Box, and zf-Ring.

In plants, the JmjC domain-containing genes are mainly involved in plant developmental processes such as flowering transition and rhythm-related processes^16^. AtJMJ12/REF6 is the first reported H3K27me2/3 demethylase in plants^17^. AtJMJ12/REF6 and AtJMJ11/ELF6 were found to interact with the transcription factor BES1 in the BR signaling pathway, suggesting that histone demethylases can exert function by recruiting sequence-specific transcription factors^18^. AtJMJ30 can act directly on the H3K27me3 demethylation of the FLC region. The double mutant of AtJMJ30 and AtJMJ32 flowers early at higher temperatures, while heterologous expression of AtJMJ30 flowers late^19^. In addition, JmjC domain-containing genes have been shown to be regulated by miRNAs. In different models of Ras-induction and tumor formation in zebrafish, Viviana et al. found that two Ras-induced microRNAs (miR-146a and 193a) target JmjD6, inducing downregulation of its mRNA and protein levels at the onset of Ras expression during melanoma development^20^.

Jatropha (*Jatropha curcas* L.) is a small perennial tree of the Euphorbiaceae family with high oil content (40–50%) in its seeds. It is drought and salt tolerant, and has a wide range of adaptability under various agro-climatic conditions. In view of its great potential for biofuel production, as well as the gradual depletion of fossil energy resources and increasing costs, the research on Jatropha is now attracting extensive attention^21,22^. However, there are few studies on the identification and function of JmjC domain-containing histone demethylase gene family in Jatropha (JcJMJ genes). In this study, we performed a comprehensive analysis of JcJMJ genes, including their phylogenetic relationships, gene structure, motif and domain composition, chromosome location, gene duplication and interspecies co-collinearity, cis-acting and miRNA recognition elements, and expression profiles, which laid the foundation for further studies on the biological functions of JcJMJs gene in the Jatropha.

## Results

### Identification of family members of the JmjC domain-containing gene in *J. curcas* L

Using a combinatorial approach, we identified 20 JmjC domain-containing genes in *J. curcas* (the same number of 20 for both HMM and Blastp methods). We found that the number of JmjC domain-containing gene in different species tend to be conserved, with the numbers of JmjC domain-containing genes in Arabidopsis, rice, and maize being 21, 20, and 20, respectively.

Basic information about the JcJMJs, such as gene length, isoelectric point (pI), and molecular weight (Mw), are calculated (listed in Table S1). The identified JcJMJ genes encode proteins ranging from 363aa (JcJMJ14) to 2442aa (JcJMJ09), with pI ranging from 4.83 (JcJMJ16) to 8.58 (JcJMJ08) and Mw ranging from 41.02 kDa (JcJMJ14) to 276.62 kDa (JcJMJ09). Notably, the length showed a trimorphic distribution (Fig. S1). The length of short size group is 363-786aa with an average length of 517aa, the medium size group is 875-1312aa with an average length of 1049aa, and the long size group is more than 1475aa with an average length of 1758aa. The number of genes in the short, medium, and long size groups were 6, 9, and 5, respectively.

### Phylogenetic analysis of the JmjC domain-containing gene in *J. curcas* L

**Figure 1:**
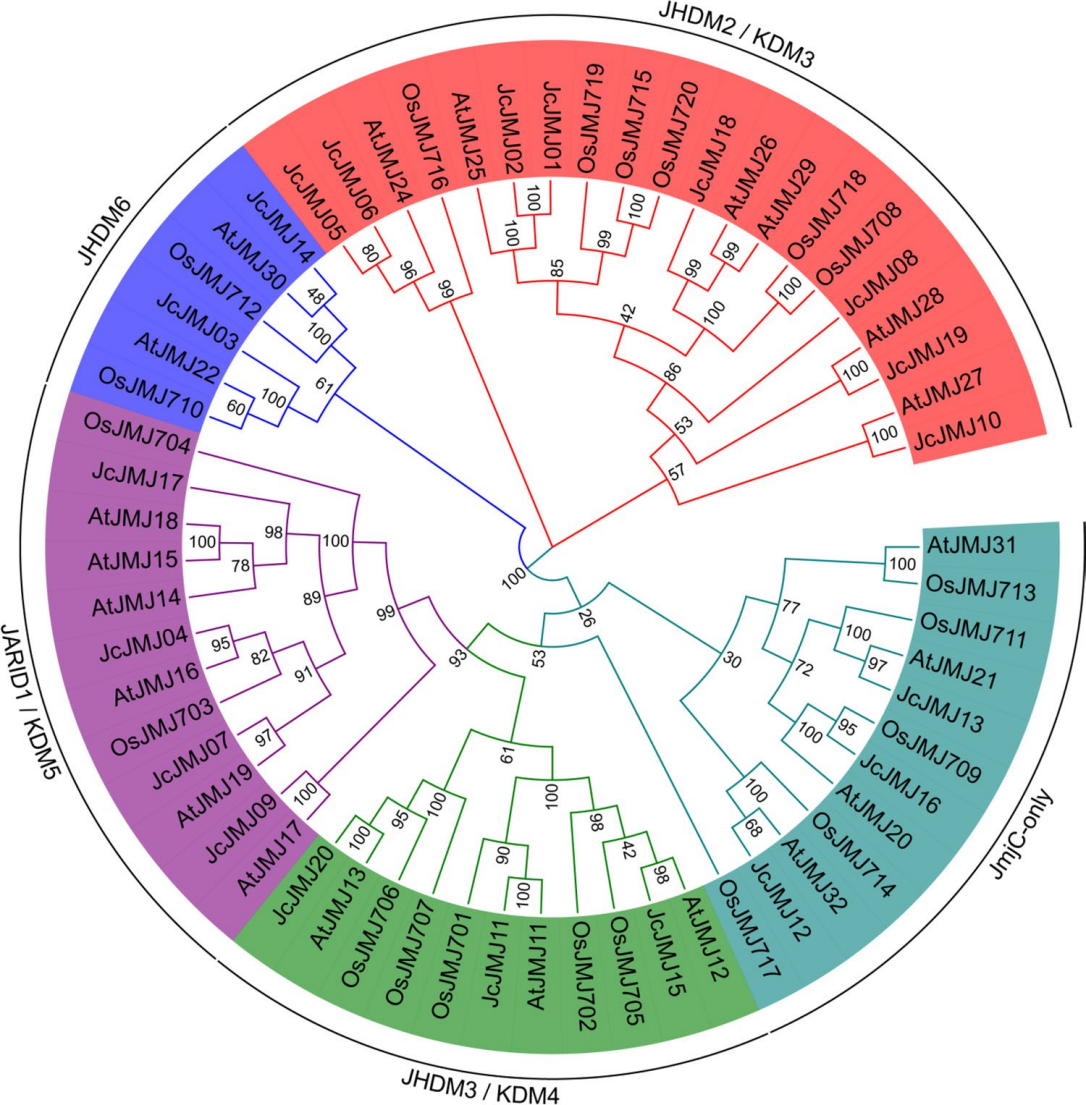
Phylogenetic relationship of JMJ genes in *J. curcas*, *Oryza sativa* and *Arabidopsis thaliana*. JMJ genes are clustered into five groups which are marked by different colors. The bootstrap values are marked on the nodes with omitted “%”.

Phylogenetic tree was constructed using the protein sequences of 21 published AtJMJs, 20 OsJMJs, and 20 identified JcJMJs (Fig. 1). Based on the comparison and analysis of JmjC domain-containing genes diversity and phylogeny, these proteins were classified into five major subfamilies: (I) JARID1/KDM5, (II) JHDM3/KDM4, (III) JHDM2/KDM3, (IV) JHDM6/JMJD6, and (V) JmjC-only, with each subfamily of *J. curcas* containing 4, 3, 8, 3, and 2 identified JmjC domain-containing genes, respectively. We found that JMJ gene sequences are relatively conservative, and the corresponding genes in *J. curcas*, *Arabidopsis thaliana* and rice are evenly distributed in each subfamily and distributed in clusters, without species-specific branches. It is noteworthy that the genes in each subfamily also have some preference in length: two subfamilies, group IV and V, prefer to have JMJ genes in the short size range, two subfamilies, group I and II, prefer to have JMJ genes in the medium size and long size ranges, and the JMJ genes in subfamily III are distributed in all three size ranges.

### Extra domain analysis of the JmjC domain-containing gene in *J. curcas* L

**Figure 2:**
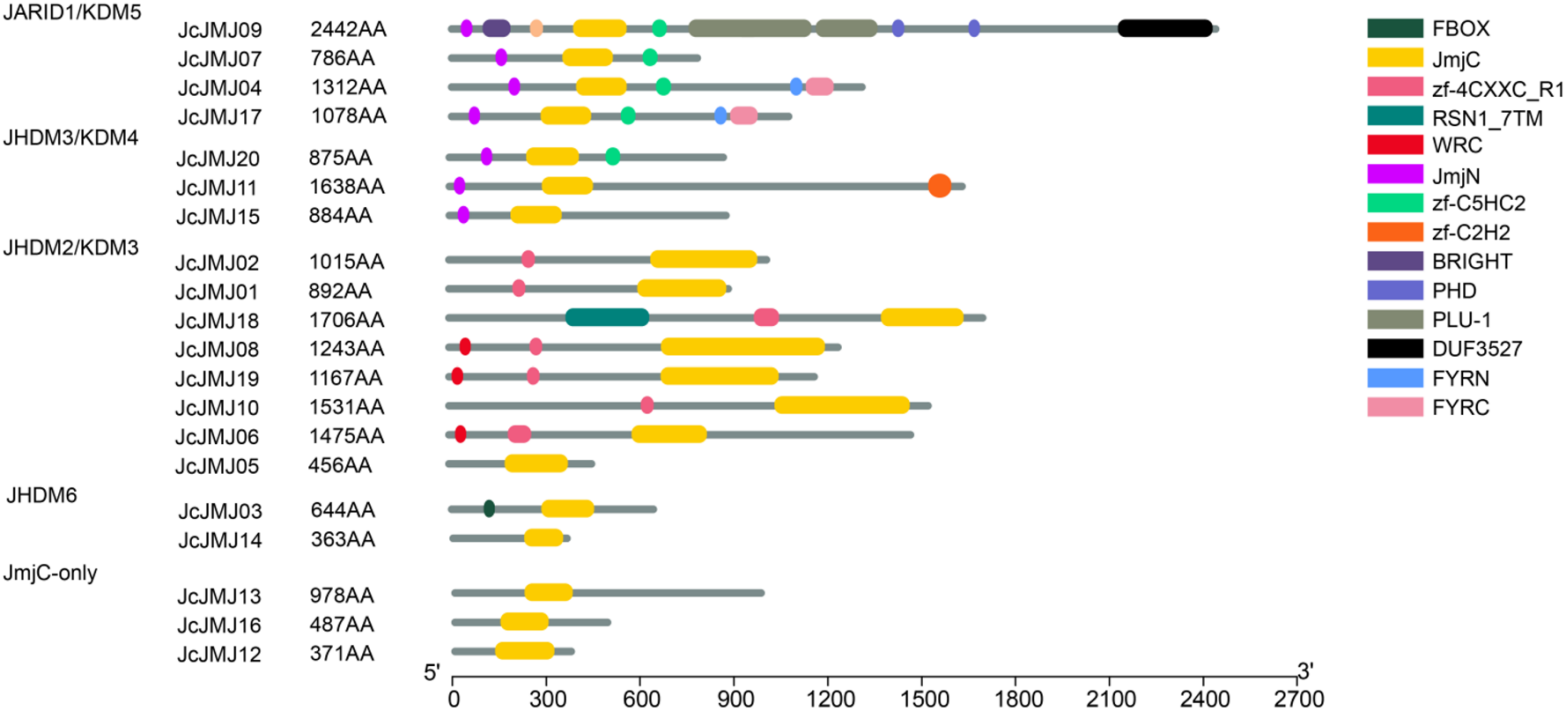
Schematic structure of JmjC domain-containing histone demethylase gene family in *J. curcas* L. Schematic representation of conserved domains identified among each subfamily of JcJMJs genes. The location and size of domains are shown by different color rectangles as indicated in the key.

To further verify the phylogenetic tree grouping, we also examined the distribution of different types of functional domains in JcJMJ genes. We found that JcJMJ genes can be divided into five groups according to the distribution of different types of domains, which corresponded to the phylogenetic tree grouping (Fig. 2). In group I JARID1/KDM5 subfamily, most members share three domains: JmjC, JmjN, and zf-C5HC2. JmjN domain is the second most widespread domain, which appears in all members of two groups, namely group I JARID1/KDM5 and group II JHDM3/KDM4 subfamily. In the group III JHDM2/KDM3 subfamily, each member has a Ring domain, and the zf-Ring domain is necessary for the demethylation activity of KDM3A. In addition, two members of the group I JARID1/KDM5 subfamily contain FYRN and FYRC domains, which may have chromatin-binding activity or contribute to JmjC domain function through collaboration with other proteins^11^.

### Gene structure and motif analysis of the JmjC domain-containing gene in *J. curcas* L

**Figure 3:**
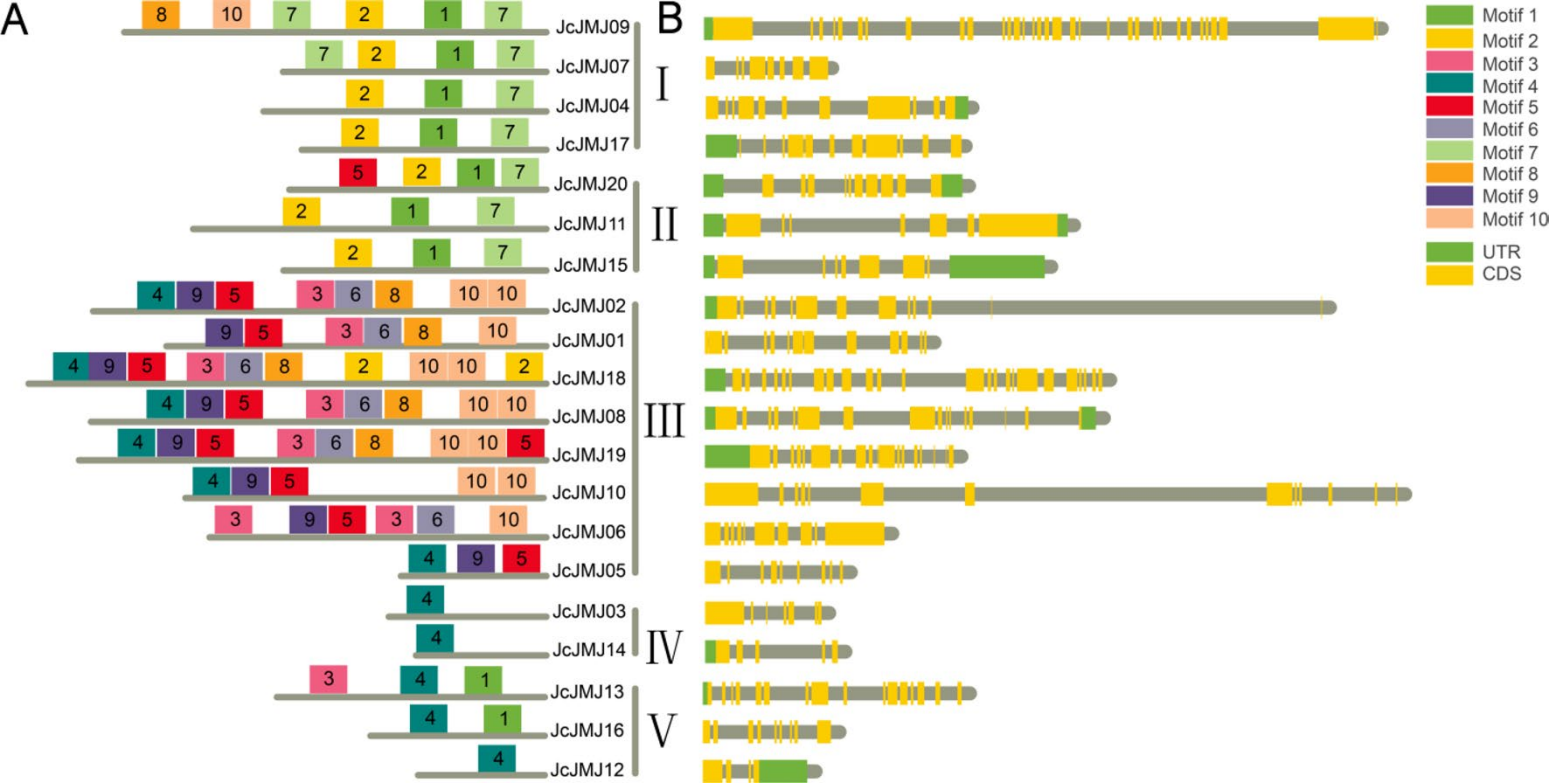
Motifs and gene structures of JcJMJ genes in *J. curcas*. (**A**) Different kinds of motifs are marked by boxes with different colors. (**B**) Gene structure of 20 JcJMJ genes. UTRs and CDSs are marked by green and yellow boxes respectively. Grey rounded rectangles represent introns.

Next, we explored the motifs and the gene structures of CDS and UTR for JcJMJ genes (Fig. 3). We found a preference for each type of motif, again validating our phylogenetic tree grouping. In Fig. 3A, group I JARID1/KDM5 and group II JHDM3/KDM4 both contain conserved motifs 1, 2 and 7. Group III JHDM2/KDM3 has the most complex motif combination, and every member except JcJMJ01 has motifs 4, 5, and 9. While the motif combinations in Group IV JHDM6/JMJD6 and Group V JmjC-only are very simple, especially the genes in Group IV has only motif 4. The detailed motif symbol and the corresponding motif consensus can be found in Fig. S2.

The CDS-UTR structure of JcJMJ genes is shown in Fig. 3B. Our analysis clearly revealed that most of the JcJMJ genes from the same subfamily share a similar gene structure. Interestingly, we found large differences in the intron–exon structure of tandem duplication (JcJMJ01 and JcJMJ02) and fragment duplication (JcJMJ04 and JcJMJ07) genes (The analysis of gene duplication is detailed in the next section). This is also consistent with the fact that the structural divergence has been very prevalent in duplicate genes and, in many cases, has led to the generation of functionally distinct paralogs^23^.

### Chromosomal localization, gene duplication and interspecies co-collinearity analysis of the JmjC domain-containing gene in *J. curcas* L

**Figure 4:**
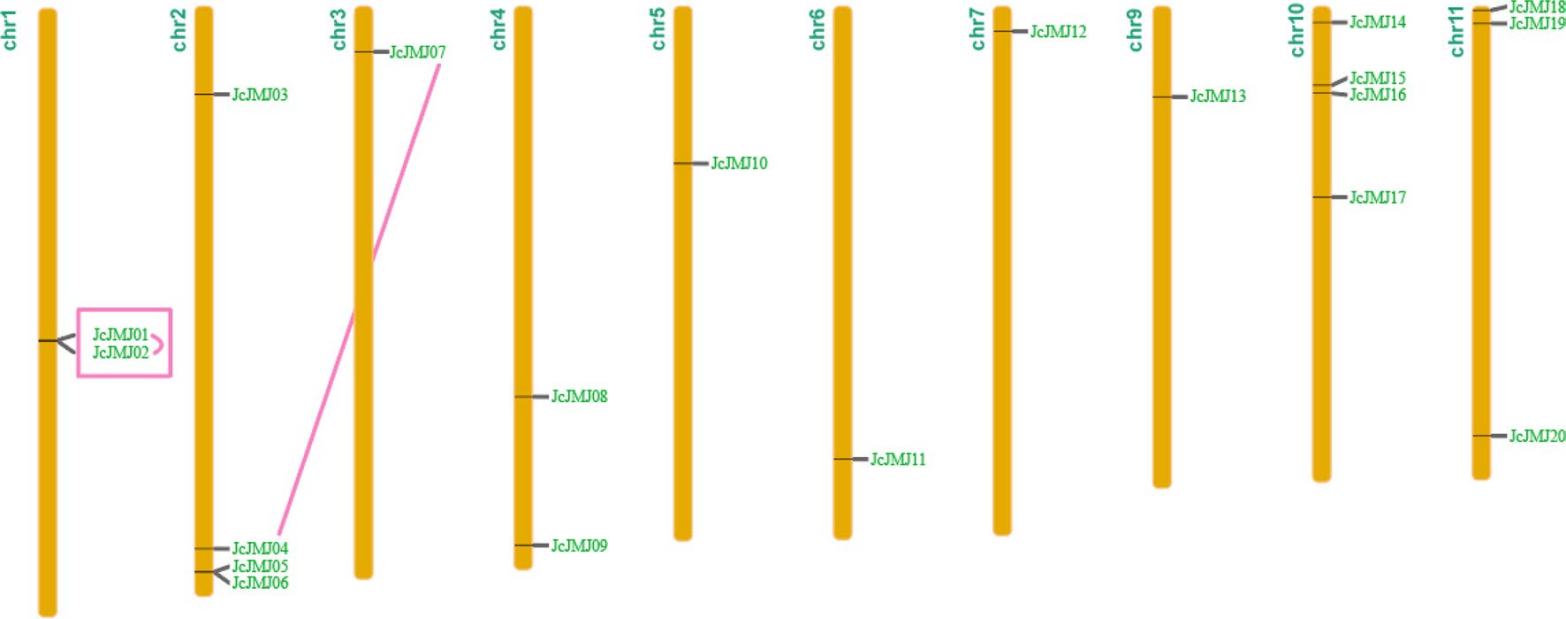
Chromosome locations and gene duplication events of JcJMJ genes in *J. curcas*. Gene names are on the right side of each chromosome according to the locations of JcJMJ genes. Segmentally duplicated genes are connected by pink line. Tandemly duplicated genes are connected by pink line and in the pink box.

To further explore the evolutionary origins and functional differentiation of the JcJMJ genes, we examined the chromosomal localization and gene duplication of the JcJMJs. 21 JcJMJs were distributed on the 10 chromosomes of *J. curcas*, and most of the JcJMJs were distributed on both ends of the chromosomes (Fig. 4). We found a pair of tandem duplications (JcJMJ01 and JcJMJ02) and a pair of segmental duplications (JcJMJ04 and JcJMJ07). All the predicted tandem and segmental duplications were found within the same subgroups, providing good support for our grouping scheme. In combination with the previous phylogenetic tree we found that both tandem duplicated genes JcJMJ01 and JcJMJ02 belong to group III JHDM2/KDM3, and they are on the same branch of the phylogenetic tree and have similar expression profiles across tissues and in the face of various stresses (The analysis of gene expression profiles is detailed in the next section). We speculate that the duplication occurred late and they are not functionally differentiated. In contrast, segmental duplicated genes JcJMJ04 and JcJMJ07, although belonging to the group I JARID1/KDM5, were located on different branches of the phylogenetic tree, and their expressions differed significantly across tissues and in the face of various stresses (The analysis of gene expression profiles is detailed in the next section). We speculate that their duplication events occurred much further back, resulting in a functional divergence that has already occurred.

To further understand the evolutionary constraints on the JcJMJs, we explored the JcJMJs gene expansion by calculating the synonymous and nonsynonymous positional substitutions of duplicate pairs and their ratios (Ka/Ks). We calculated the Ka/Ks ratios of the two duplicated JcJMJ gene pairs and found that their Ka/Ks ratios were less than 1, indicating that these JcJMJ genes underwent strong purifying selection to reduce deleterious mutations after replication (Fig. S3). These results are similar to those of previous studies in maize. This phenomenon indicates that the JmjC domains are relatively stable in plants and are highly conserved in evolution.

**Figure 5:**
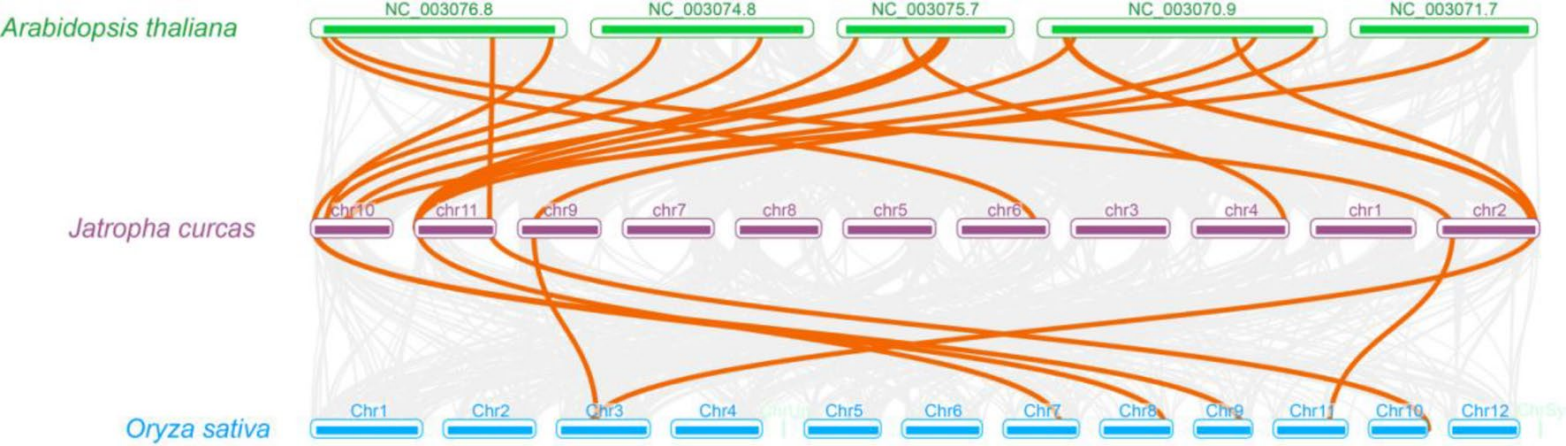
Synteny analyses of the JMJ genes among the three species *J. curcas*, *Oryza sativa* and *Arabidopsis thaliana*. The collinear blocks within *J. curcas* and other specie genomes were displayed by the gray lines. The syntenic JMJ gene pairs between *J. curcas* and other species were highlighted with the red lines.

We then examined the interspecies co-collinearity of the JMJ genes among *J. curcas*, *Oryza sativa* and *Arabidopsis thaliana* (Fig. 5). There are only seven orthologous genes of the JcJMJ genes can be found on the rice genome, whereas 15 JcJMJ genes can find their corresponding orthologous genes on the Arabidopsis genome.

### Prediction of the cis-acting elements of the JmjC domain-containing gene in *J. curcas* L

**Figure 6:**
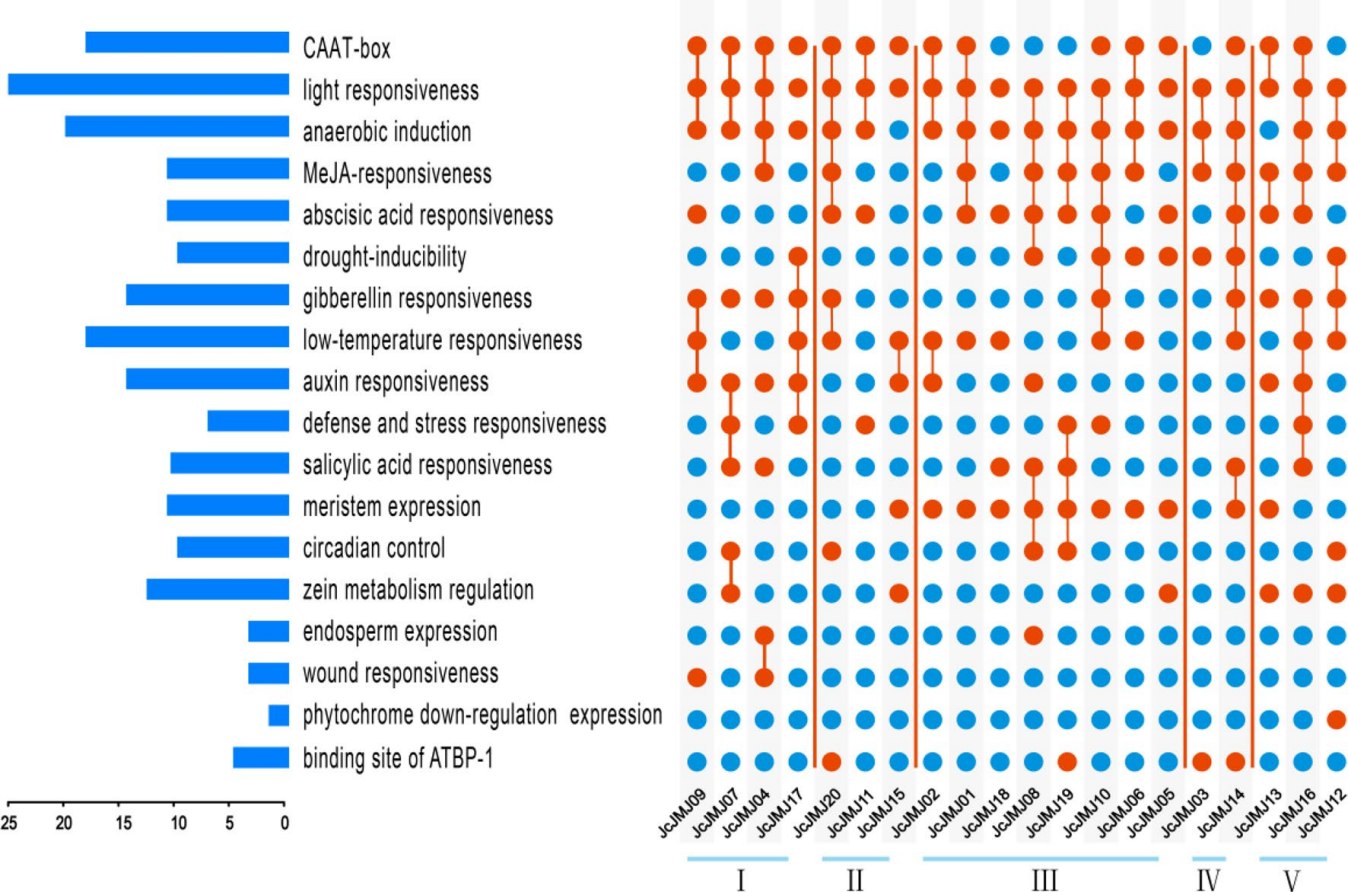
Cis-acting elements in the promoters of each JcJMJ gene. The length of the blue bar on the left indicates the number of cis-acting elements; The blue origin on the right indicates the site in the promoter of the gene that does not have the cis-acting element, while the red color indicates the site that does.

To further clarify how the JmjC domain genes, which tends to be conserved in evolution, achieve subfamily functional diversity, we elucidated the possible regulatory mechanisms of JmjC domain-containing genes in *J. curcas* in response to abiotic or biotic stresses. We used the PlantCARE database to analyze the promoter sequences of the JcJMJ genes to identify cis-regulatory elements in the promoter region. 18 types of cis-regulatory elements associated with responses to light response, gibberellin, drought, or metabolism were detected in the promoter of the JcJMJs (Fig. 6). Each JcJMJ gene contains multiple regulatory elements. Notably, the light responsiveness regulatory element was present in all members of the five subfamilies, and two regulatory elements, CAAT-box and anaerobic induction, were also predicted to be present in most members. The two regulatory elements gibberellin responsiveness and auxin responsiveness were prevalent in most members of groups I and V, and the meristem expression regulatory element, which was present in every member of group III, but rarely in the other subfamilies. Binding site of ATBP-1 regulatory element is present in both members of group IV and few other subgroups. Thus, these results demonstrate that the expression of the JcJMJ genes is regulated by various environmental factors.

### Prediction of miRNA target sites for the JmjC domain-containing gene in *J. curcas* L

**Figure 7:**
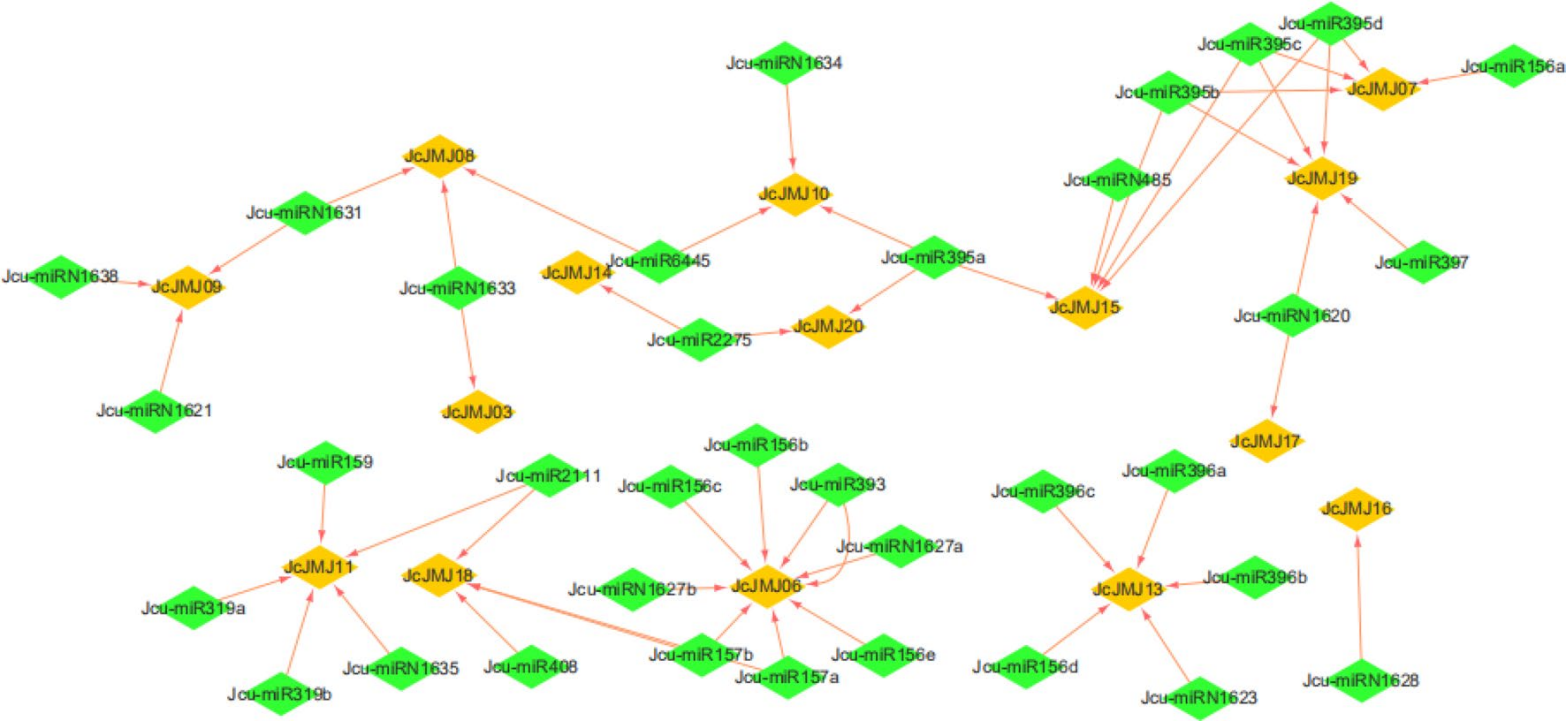
Regulatory networks between the conserved miRNAs and their targeted JcJMJ genes. The green and yellow rectangles represent miRNA and JcJMJs, respectively. The direction of the arrow indicates the direction of the target regulation.

It has been reported that two Ras-induced microRNAs (miR-146a and 193a) target JmjD6 in animals, thereby inducing down-regulation of its mRNA and protein levels at the onset of Ras expression during melanoma development^20^. Thus, although no instance of JmjC targeted by miRNA has been reported, we speculate that this phenomenon might also exist in plants. After prediction by psRNATarge, we found that JcJMJ genes may be targeted by some common conserved miRNA families, such as miRNA156, miRNA159, miRNA319, miRNA393, and miRNA395, and then form a complex miRNA-mRNA regulatory network (Fig. 7, see the Table S2 for details). We know that miRNAs are involved in the regulation of almost all aspects of plant growth and metabolism, such as leaf development and formation (miR156^24^, miRNA165/166^25^, miR319^26^), stomatal development (miR824)^27^, lateral root formation (miR164)^28^, regulation of the transition from nutritional to reproductive growth (miR172)^29^, flower development (miR172, miR159)^30^. We speculate that the regulation of the JcJMJ genes by so many important miRNAs may be an important reason for the functional diversity of its subfamilies.

Interestingly, the group III JHDM2/KDM3 subfamily was regulated by the largest number of miRNAs, up to 24 (Fig. S4). In combination with the previous JcJMJ gene stress-related expression analysis, we found that the group III JHDM2/KDM3 subfamily genes are very functionally divergent and are clustered into different classes in the expression profile. In addition, the genes in JHDM6 subfamily had only two miRNA regulatory sites in total, and the two members were clustered together in the expression profile with no significant difference. We speculate that the functional diversity of the JcJMJ genes may have some relationship with the miRNA-mediated post-transcriptional regulatory network regulating them.

### Gene expression of the JmjC domain-containing gene in *J. curcas* L

**Figure 8:**
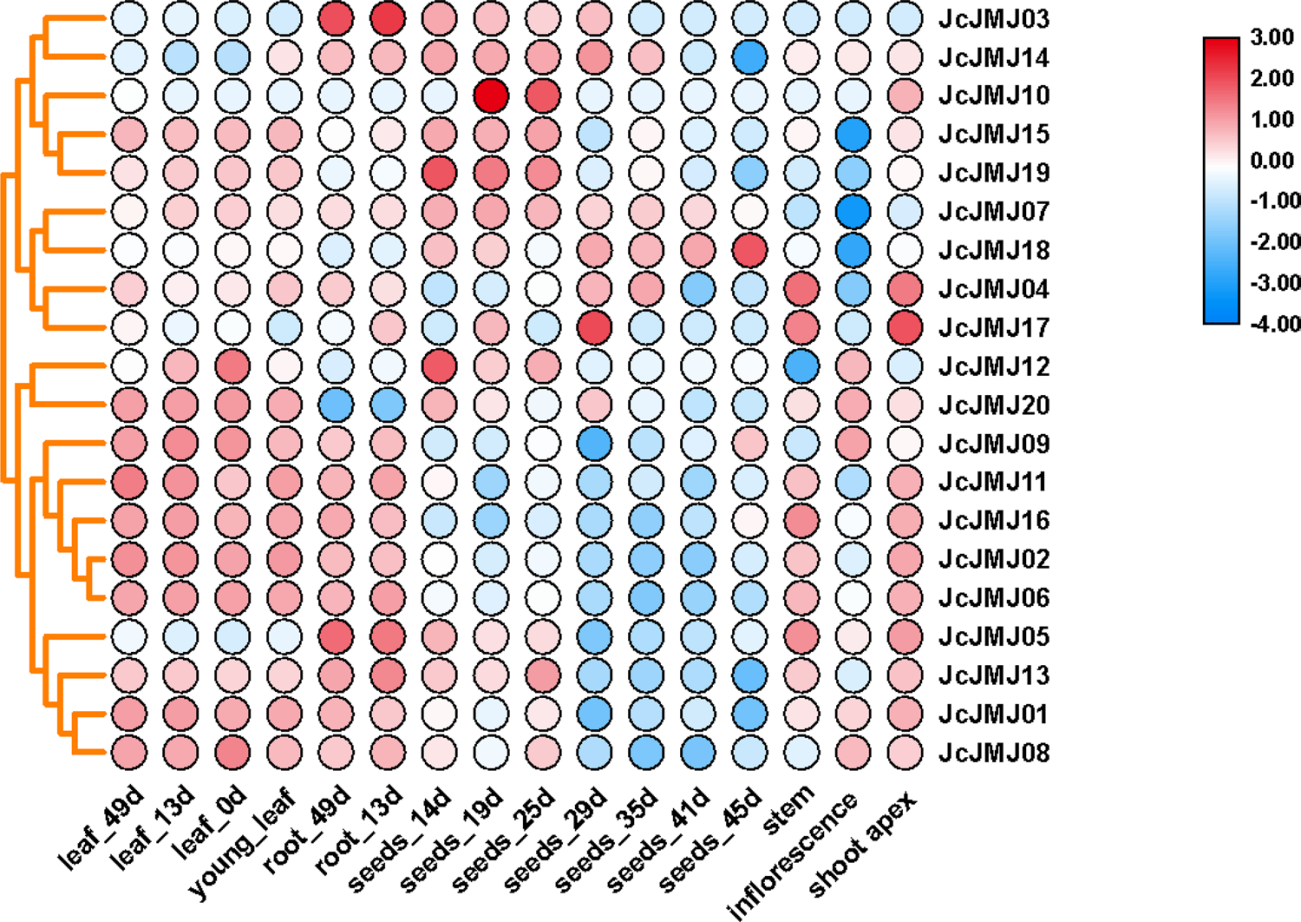
Hierarchical clustering of the expression profiles of 20 JcJMJ genes in 6 tissues. The color scale representing log2 signal values is shown on the right. Blue represents a low level and red indicates a high level of transcript abundance. The different tissues and/or organs are noted on the bottom of each lane. Cluster dendrograms by row scale are shown on the left.

To further investigate the possible functions of JcJMJs in plant growth and development, we analyzed the expression data of JcJMJs in stem, inflorescence, bud, leaf, root, and seed of *J. curcas*. From the heat map (Fig. 8), it can be seen that all 20 JcJMJs were expressed at different levels in six tissues at different developmental stages. While most members of group II JHDM3/KDM4 (except JcJMJ15) and group V JmjC-only (except JcJMJ12) were expressed at higher levels in leaves and roots. And the members of group I JARID1/KDM5 (except JcJMJ07) were highly expressed in stems and stem tips.

In addition, some tissue/organ-specific genes were identified, such as JcJMJ10, a member of group III JHDM2/KDM3, to be highly expressed in seeds collected at 19 and 25 days after pollination, but hardly expressed in other tissues of *J. curcas*. JcJMJ18 was consistently expressed at a high level in seeds, but almost non-expressed in other tissues.

**Figure 9:**
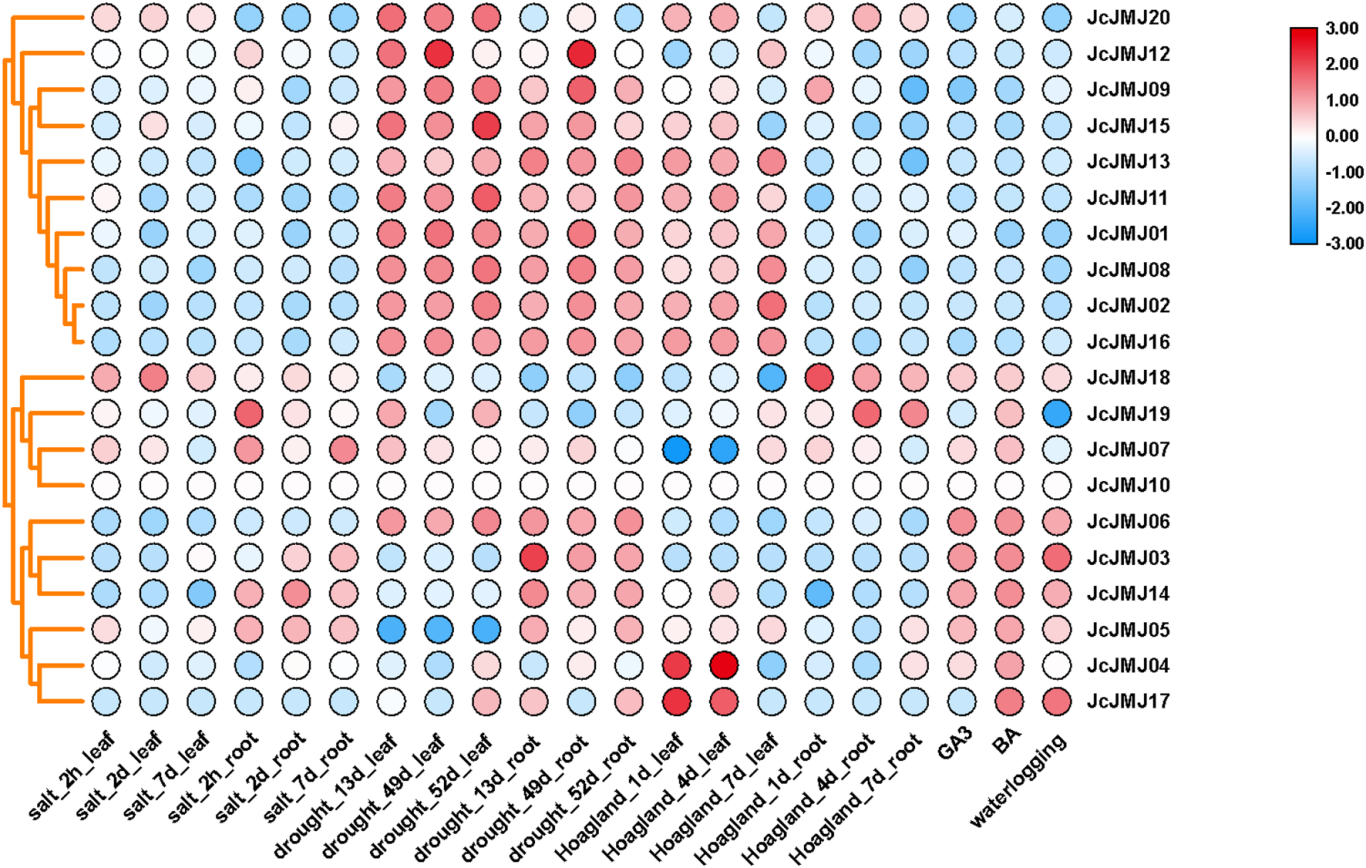
Expression patterns of JcJMJ genes under different treatments. Normalized log2 transformed values were used with hierarchical clustering represented by the color scale (− 3 to 3). Blue indicates low expression, and red indicates high expression. Cluster dendrograms by row scale are shown on the left.

We also studied the expression profiles of the JcJMJ genes under different stresses (Fig. 9). We can see that the expression of JcJMJ10 gene remained unchanged in all treatments. In combination with the tissue-specific expression profiles in Fig. 8, we can see that it is because JcJMJ10 gene was only highly expressed in seeds collected at 19 and 25 days after pollination, while the expression levels were barely detectable in other tissues. Therefore, we could not detect any changes in expression in these tissues after various stress treatments. Almost all members of JmjC-only genes (JcJMJ12, JcJMJ13, and JcJMJ16) showed decreased expression in leaves and roots under salt stress, Hoagland's nutrient solution, gibberellic acid, 6-benzylaminopurine, and water immersion treatments. Moreover, almost all the expressions in leaves and roots of these genes showed an increased expression under drought treatment. In leaves under salt stress treatment, only the expression of JcJMJ18 increased significantly, while the expression of the other JcJMJ genes decreased or increased slightly (JcJMJ05, JcJMJ20, JcJMJ15 and JcJMJ07), among which the expression of JcJMJ14 decreased most significantly. Under gibberellin treatment, the expression of two JcJMJ genes (JcJMJ03 and JcJMJ06) more than doubled. Under 6-benzylaminopurine treatment, the expression of all members of Group IV JHDM6 (JcJMJ3 and JcJMJ14) increased significantly. These results further demonstrate that JcJMJ genes exhibit different expression patterns under different environmental stress conditions, suggesting that these genes are responsive to stress treatments.

## Discussion

Histone methylation plays an important role in the epigenetic regulation of gene expression, which is determined by the internal stabilization of histone methylation regulated by histone methyltransferases and demethylases. JmjC domain proteins represent a large family of histone demethylases in animals and plants, which play an important role in histone modification and are important components of epigenetics. However, the identification and functional studies on the histone demethylase gene family in *J. curcas* are still scarce. In this study, 20 non-redundant JcJMJs genes were identified and characterized from the latest version of *J. curcas* genome. A series of comprehensive analysis was carried out, such as phylogenetic relationships, conserved domains, gene structure and motif, chromosome position and duplication, cis-acting elements and miRNA target sites, tissue expression and stress expression. All these analyses laid the foundation for further study of the biological function of the JcJMJ genes.

In our study of the JcJMJs, we found that the JcJMJ genes are mainly divided into five different subclasses, which are also present in Arabidopsis and rice. The genome size of the *J. curcas* is about 375 Mbp, which is three times bigger than the Arabidopsis (about 125 Mbp) genome and slightly smaller than the rice (about 389 Mbp) genome. Twenty JmjC domain-containing genes were identified in the *J. curcas*, which is only one less than AtJMJs (21) and equal in nu41mber to OsJMJs (20). Through a review of papers, we found the number of JmjC domain genes in maize, strawberry, grape, and lotus to be 19, 20, 20, and 20, respectively. One exception is soybean, which has 48 JmjC domain-containing genes. This should be because soybeans have gone through two rounds of whole genome duplication (WGD), which may have led to the unusual amplification of JmjC gene families. This phenomenon suggests that the JmjC domain is relatively stable in plants, is highly conserved in evolution, and has little to do with genome size. However, the functions of the JMJ genes are definitely very diverse, and even genes of the same subfamily are highly divergent in function.

To further explore the causes to the JcJMJ genes’ functional diversity, we examined the cis-acting and miRNA recognition elements related to JcJMJ genes, and their expression profiles in different tissues and stress treatments. We detected 18 types of cis-regulatory elements associated with responses to light response, gibberellin, drought, or metabolism in the promoter of the JcJMJ genes. The number and type of cis-regulatory elements in the promoter regions of the group III JHDM2/KDM3 subfamily genes varied widely, which we speculate may account for the functional diversity of this subfamily of genes. In addition, we found that the JcJMJ genes have been regulated by numerous conserved miRNA families, such as miR156, miR159, miR319, miR393 and miR395. Among them, the group III JHDM2/KDM3 subfamily, which is more differentiated, has the largest number of miRNA target sites. We know that overexpression of miR156 can increase the formation of Arabidopsis leaves, making the apical dominance less pronounced while delaying flowering^31^. miR159 and miR319, two miRNAs with very similar nucleotide sequences, can both recognize and act together on the MYB and TCP transcription factor families, which play an important regulatory role in leaf morphogenesis^32^. miR393 expression is up-regulated under drought, low temperature, salt, and ABA treatment conditions; miR395 expression levels are elevated in the absence of sulfate. Therefore, these conserved miRNA families that regulate JcJMJ genes may contribute a lot to the functional diversity of JcJMJ gene family^33^.

What’s more, by studying the expression profiles in different tissues and stress treatments of the JcJMJ genes, we found that the JcJMJ genes exhibit different expression patterns in response to different types of stresses at different developmental stages. For example, we found that most of the JcJMJ genes from group II JHDM3/KDM4 and group V JmjC-only had significantly higher expression when subjected to drought stress, suggesting that these two subfamilies are closely related to the response to drought stress. Members of the group I JARID1/KDM5 subfamily had reduced expression in response to most stresses. However, the expression of JcJMJ4 increased in response to BA and waterlogging treatments, suggesting that JcJMJ4 may be closely related to BA and waterlogging stresses. These diverse gene expression patterns indicate the functional diversity of JcJMJ genes. Besides, under abiotic stress treatment, some genes showed contradictory expression patterns, which we speculate may be related to the fact that these genes exhibit specificity in different tissues, which may suggest a potential mechanism for JcJMJ genes that needs to be further investigated in the future. Our cis-acting and miRNA recognition elements analysis suggested that this diversity should come from the diverse transcriptional and post-transcriptional regulation of the JcJMJ genes, rather than the differentiation of the gene sequences. This is an interesting speculation. However, it is only based on the analysis of JmjC genes in *J. curcas*. It is necessary to conduct research in the whole Euphorbiaceae or even more green plants from different families, and to further validate our bioinformatics analysis through more experiments.

## Methods

### Identification and analysis of physicochemical properties of JmjC genes in *J. curcas* L

To identify the JmjC domain-containing genes of *J. curcas*, we used both genome-wide Hidden Markov Model^34^ (HMM) search and BLASTP^35^ comparison to mutually validate the predictions. The Hidden Markov Model PF02373 was downloaded from Pfam Database^36^. 21 and 20 JmjC domain-containing protein sequences published in Arabidopsis and rice, were used as initial query sequences and searched using BLASTP. All sequences obtained by both methods were further confirmed using the NCBI Conserved Domain Database^37^ (CDD) with default parameters and the SMART^38^ online analysis program. The resulting candidate genes were used to calculate the physicochemical parameters of each gene product, including molecular weight (KDa) and isoelectric point (pI), using ExPASy's^39^ pI/Mw tool with default parameters.

### Phylogenetic analysis of the JmjC domain-containing gene in *J. curcas* L

Multiple sequence alignment of all predicted JmjC domain-containing protein sequences of *J. curcas* with their orthologs from Arabidopsis and rice was performed using Muscle^40^ under Linux. All protein sequences were downloaded from the National Center for Biotechnology Information (NCBI). Then, based on this alignment, sequences were clipped aligned using trimAl^41^, the optimal model was found using ModelFinder and build the tree using iqtree^42^, and finally use Evolview to visualize and beautify the tree file^43^.

### Extra domain analysis of the JmjC domain-containing gene in *J. curcas* L

We knew about that JmjC domain-containing genes usually have other conserved domains, so we used a search function called Batch Conserved Domain from NCBI to further search for other domains of the JmjC domain-containing gene in the *J. curcas*.

### Gene structure and motif analysis of the JmjC domain-containing gene in *J. curcas* L

All protein sequences of JmjC domain-containing genes were searched using the MEME^44^ online tool for motifs other than the JmjC domain, which are located outside the JmjC domain and are conserved. The chromosomal location information of JcJMJ genes and their CDS and UTR regions was obtained from the *J. curcas* genome annotation file, and the gene structure was drawn using the GSDS2.0 Gene Structure Display Server)^45^.

### Chromosomal localization, gene duplication and interspecies co-collinearity analysis of the JmjC domain-containing gene in *J. curcas* L

Based on the chromosomal location of the genes, we used MapInspect to map the distribution of the JmjC domain-containing genes. Duplicate gene pairs were obtained from tandem or fragmented repeats according to methods described in the Plant Genome Repeat Database^46^. An all-against-all BLASTP comparison (e-value ≤ 1e−10) provided gene pairs for syntenic clustering using MCScanX^47^ (e-value ≤ 1e−10). Segment duplication was also predicted by the micro-fragment comparison method. The JmjC duplicate gene pairs from the above analysis were further examined by BLASTP (e-value ≤ 1e−10), and all the JmjC genes obtained from the above analysis were used as anchors of micro-fragments generated by the collection of 10 upstream and 10 downstream coding genes. Tandem duplications were identified if two JmjC genes were next to each other or they had one unrelated gene between them. The JcJMJ gene pairs generated from the fragments or tandem duplicates were marked with pink linear linkages and pink rectangles, respectively.

To further explore the synchronous relationship between the JmjC domain-containing genes in the *J. curcas* and the homologous JmjC genes in other species, we additionally downloaded genomic data and gene annotation files from Phytozome for Arabidopsis and rice (*Oryza sativa*), and did a collinearity analysis of JmjCs genes among the *J. curcas*, Arabidopsis, and rice.

### Prediction of the cis-acting elements of the JmjC domain-containing gene in *J. curcas* L

A 2 kb sequence upstream of the start codon (ATG) of each JcJMJ gene was taken and the PlantCARE^48^ database was used to search for stress response and hormone-related cis-acting elements in the promoter sequence of the JmjC-containing domain genes.

### Prediction of miRNA target sites for the JmjC domain-containing gene in *J. curcas* L

The mature sequences of all currently cataloged members of the *J. curcas* miRNA family were obtained from Plant miRNA Encyclopedia^49^, and the identified *J. curcas* JmjC-containing domain genes were used as target genes for miRNA target prediction analysis using the psRNATargete^50^. We used cytoscape v3.7.1^51^ to map the network of each subfamily of JmjC-containing domain genes, regulated by the number of miRNAs.

### Gene expression profiling of the JmjC domain-containing gene in *J. curcas* L

To determine the expression pattern of identified JmjC domain-containing genes in the tissues of the *J. curcas*, we examined the expression of the JcJMJs gene in the *J. curcas* through public transcriptomic data. Raw expression data of different tissues including seeds, roots, leaves, stems, inflorescence meristems, and stem tips were obtained by searching the NCBI SRA database. We also took raw expression data of tissues subjected to different treatments, including gibberellic acid [GA], 6-benzylaminopurine [BA], high salt concentration and drought, and nutrient solution (Hoagland) (Table S3). Transcriptome analysis of gene expression by RNA sequencing (RNA-seq) using the HISAT2-Stringtie-FeatureCounts RNASeq pipeline. The specific parameters can be found in the attached Table S4. We have integrated our pipeline using snakemake^52^. Finally, tissue and stress expression profiles of JcJMJs were generated using the pheatmap software package in the R v.3.6.1.

## Conclusion

A total of 20 JcJMJ genes were identified in this study, distributed on 10 chromosomes. These JcJMJ genes were mainly divided into five subfamilies based on amino acid sequence similarity. The gene structures, distribution of conserved domains and motifs were fairly similar among members of the same subfamilies. The prediction of the miRNA target sites of JcJMJ genes revealed that JcJMJ genes may be regulated by a complicated miRNA-mediated post-transcriptional regulatory network. In addition, the expression profiles of JcJMJ genes in different tissues and stress treatments indicated that many JcJMJ genes play functional developmental roles in different tissues, and exhibit significant differential expression under different stress treatments. Taken together, these findings provide valuable clues for further investigation of the specific gene function and gene diversity of JmjC gene family in *J. curcas* L. and other plants.

## Supplementary Information

Supplementary Information 1.

Supplementary Information 2.

## Data Availability

The datasets supporting the conclusions of this article are included in the article and in its additional files.

## References

[1] Cigliano RA (2013). Histone deacetylase AtHDA7 is required for female gametophyte and embryo development in Arabidopsis. Plant Physiol..

[2] Badeaux A, Shi Y (2013). Emerging roles for chromatin as a signal integration and storage platform. Nat. Rev. Mol. Cell Biol..

[3] Al-Shyoukh I (2011). Systematic quantitative characterization of cellular responses induced by multiple signals. BMC Syst. Biol..

[4] Holliday R (1987). DNA methylation and epigenetic defects in carcinogenesis. Mutat. Res..

[5] Klose RJ, Kallin EM, Zhang Y (2006). JmjC-domain-containing proteins and histone demethylation. Nat. Rev. Genet..

[6] Klose RJ, Zhang Y (2007). Regulation of histone methylation by demethylimination and demethylation. Nat. Rev. Mol. Cell Biol..

[7] Lu F, Cui X, Zhang S, Liu C, Cao X (2010). JMJ14 is an H3K4 demethylase regulating flowering time in Arabidopsis. Cell Res..

[8] Allis CD (2007). New nomenclature for chromatin-modifying enzymes. Cell.

[9] Ahmad A, Cao X (2012). Plant PRMTs broaden the scope of arginine methylation. J. Genet. Genomics.

[10] Zhang L, Ma H (2012). Complex evolutionary history and diverse domain organization of SET proteins suggest divergent regulatory interactions. New Phytol..

[11] Lu F (2008). Comparative analysis of JmjC domain-containing proteins reveals the potential histone demethylases in Arabidopsis and rice. J. Integr. Plant Biol..

[12] Trewick SC, McLaughlin PJ, Allshire RC (2005). Methylation: lost in hydroxylation?. EMBO Rep..

[13] Chen X, Hu Y, Zhou DX (1809). Epigenetic gene regulation by plant Jumonji group of histone demethylase. Biochim. Biophys. Acta.

[14] Tsukada Y (2006). Histone demethylation by a family of JmjC domain-containing proteins. Nature.

[15] Luo M, Hung F-Y, Yang S, Liu X, Wu K (2014). Histone lysine demethylases and their functions in plants. Plant Mol. Biol. Rep..

[16] Accari SL, Fisher PR (2015). Emerging roles of JmjC domain-containing proteins. Int. Rev. Cell Mol. Biol..

[17] Noh B (2004). Divergent roles of a pair of homologous jumonji/zinc-finger–class transcription factor proteins in the regulation of Arabidopsis flowering time. Plant Cell.

[18] Yu X (2008). Modulation of brassinosteroid-regulated gene expression by Jumonji domain-containing proteins ELF6 and REF6 in Arabidopsis. Proc. Natl. Acad. Sci. USA.

[19] Lu SX (2011). The Jumonji C domain-containing protein JMJ30 regulates period length in the Arabidopsis circadian clock. Plant Physiol..

[20] Anelli V (2018). Ras-induced miR-146a and 193a target Jmjd6 to regulate melanoma progression. Front. Genet..

[21] Bhasanutra R, Sutiponpeibun S (1982). *Jatropha curcas* oil as a substitute for diesel engine oil. Int. Energy J..

[22] Openshaw K (2000). A review of Jatropha curcas: An oil plant of unfulfilled promise. Biomass Bioenergy.

[23] Lynch M, Conery JS (2000). The evolutionary fate and consequences of duplicate genes. Science.

[24] Schwab R (2005). Specific effects of microRNAs on the plant transcriptome. Dev. Cell.

[25] Kidner CA (2010). The many roles of small RNAs in leaf development. J. Genet. Genomics.

[26] Palatnik JF (2007). Sequence and expression differences underlie functional specialization of Arabidopsis microRNAs miR159 and miR319. Dev. Cell.

[27] Kutter C, Schöb H, Stadler M, Meins F, Si-Ammour A (2007). MicroRNA-mediated regulation of stomatal development in Arabidopsis. Plant Cell.

[28] Xie Q (2002). SINAT5 promotes ubiquitin-related degradation of NAC1 to attenuate auxin signals. Nature.

[29] Chen X (2004). A microRNA as a translational repressor of APETALA2 in Arabidopsis flower development. Science.

[30] Achard, P., Herr, A., Baulcombe, D. C. & Harberd, N. P. Modulation of floral development by a gibberellin-regulated microRNA (2004). 10.1242/dev.01206 15226253

[31] Schwab R (2005). Specific effects of microRNA on the plant transcriptome. Dev. Cell.

[32] Palatnik JF (2007). Sequence and expression differences underlie functional specialization of Arabidopsis microRNAs miR159 and miR319. Dev Cell.

[33] Chiou TJ (2006). Regulation of phosphate homeostasis by microRNA in Arabidopsis. Plant Cell.

[34] Schuster-Bockler, B. & Bateman, A. An introduction to hidden Markov models. *Curr. Protoc. Bioinformatics* **Appendix 3**, Appendix 3A. 10.1002/0471250953.bia03as18 (2007). 10.1002/0471250953.bia03as18 18428778

[35] Mount, D. W. Using the Basic Local Alignment Search Tool (BLAST). *Csh Protocols* **14**, pdb.top17. (2007). 10.1101/pdb.top17 21357135

[36] Finn RD (2016). The Pfam protein families database: towards a more sustainable future. Nucleic Acids Res..

[37] Marchler-Bauer A (2015). CDD: NCBI's conserved domain database. Nucleic Acids Res..

[38] Letunic I, Doerks T, Bork P (2012). SMART 7: Recent updates to the protein domain annotation resource. Nucleic Acids Res..

[39] Gasteiger E (2003). ExPASy: The proteomics server for in-depth protein knowledge and analysis. Nucleic Acids Res..

[40] Edgar RC (2004). MUSCLE: Multiple sequence alignment with high accuracy and high throughput. Nucleic Acids Res..

[41] Capella-Gutierrez S, Silla-Martinez JM, Gabaldon T (2009). trimAl: A tool for automated alignment trimming in large-scale phylogenetic analyses. Bioinformatics.

[42] Minh BQ (2020). Corrigendum to: IQ-TREE 2: New models and efficient methods for phylogenetic inference in the genomic era. Mol. Biol. Evol..

[43] He Z (2016). Evolview v2: an online visualization and management tool for customized and annotated phylogenetic trees. Nucleic Acids Res..

[44] Bailey TL, Johnson J, Grant CE, Noble WS (2015). The MEME suite. Nucleic Acids Res..

[45] Hu B (2015). GSDS 2.0: An upgraded gene feature visualization server. Bioinformatics.

[46] Lee TH, Tang H, Wang X, Paterson AH (2013). PGDD: A database of gene and genome duplication in plants. Nucleic Acids Res..

[47] Wang Y (2012). MCScanX: A toolkit for detection and evolutionary analysis of gene synteny and collinearity. Nucleic Acids Res..

[48] Lescot M (2002). PlantCARE, a database of plant cis-acting regulatory elements and a portal to tools for in silico analysis of promoter sequences. Nucleic Acids Res..

[49] Guo Z (2020). PmiREN: A comprehensive encyclopedia of plant miRNAs. Nucleic Acids Res..

[50] Dai X, Zhuang Z, Zhao PX (2018). psRNATarget: A plant small RNA target analysis server (2017 release). Nucleic Acids Res..

[51] Smoot ME, Ono K, Ruscheinski J, Wang PL, Ideker T (2011). Cytoscape 2.8: New features for data integration and network visualization. Bioinformatics.

[52] Köster J, Rahmann S (2012). Snakemake—A scalable bioinformatics workflow engine. Bioinformatics.

