# Supplementary material for: Genome-wide identification, classification and expression analysis of the JmjC domain-containing histone demethylase gene family in Jatropha curcas L
Source: Sci Rep. 2022 Apr 21;12:6543. doi: 10.1038/s41598-022-10584-3 (PMC9023485; doi:10.1038/s41598-022-10584-3)
Supplement: Supplementary file 2 — Supplementary Information 2. [file 41598_2022_10584_MOESM2_ESM.docx]

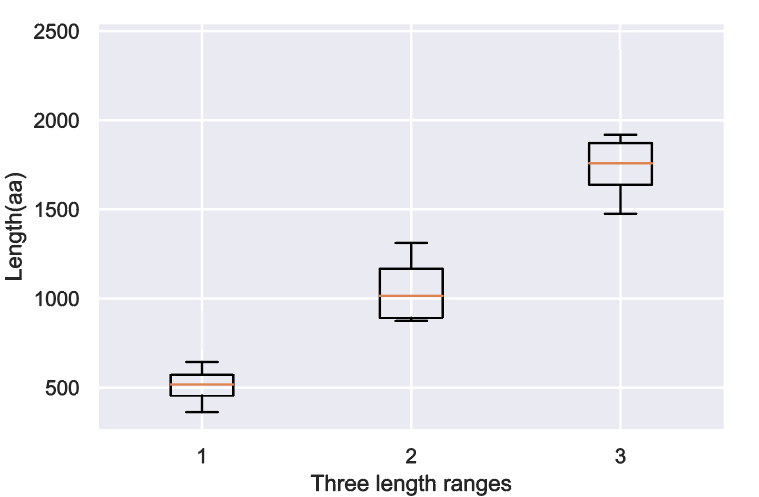


**Figure S1.** The distribution of three length ranges of JcJMJ genes. Y-axis represents protein length (aa); X-axis lists three length ranges.


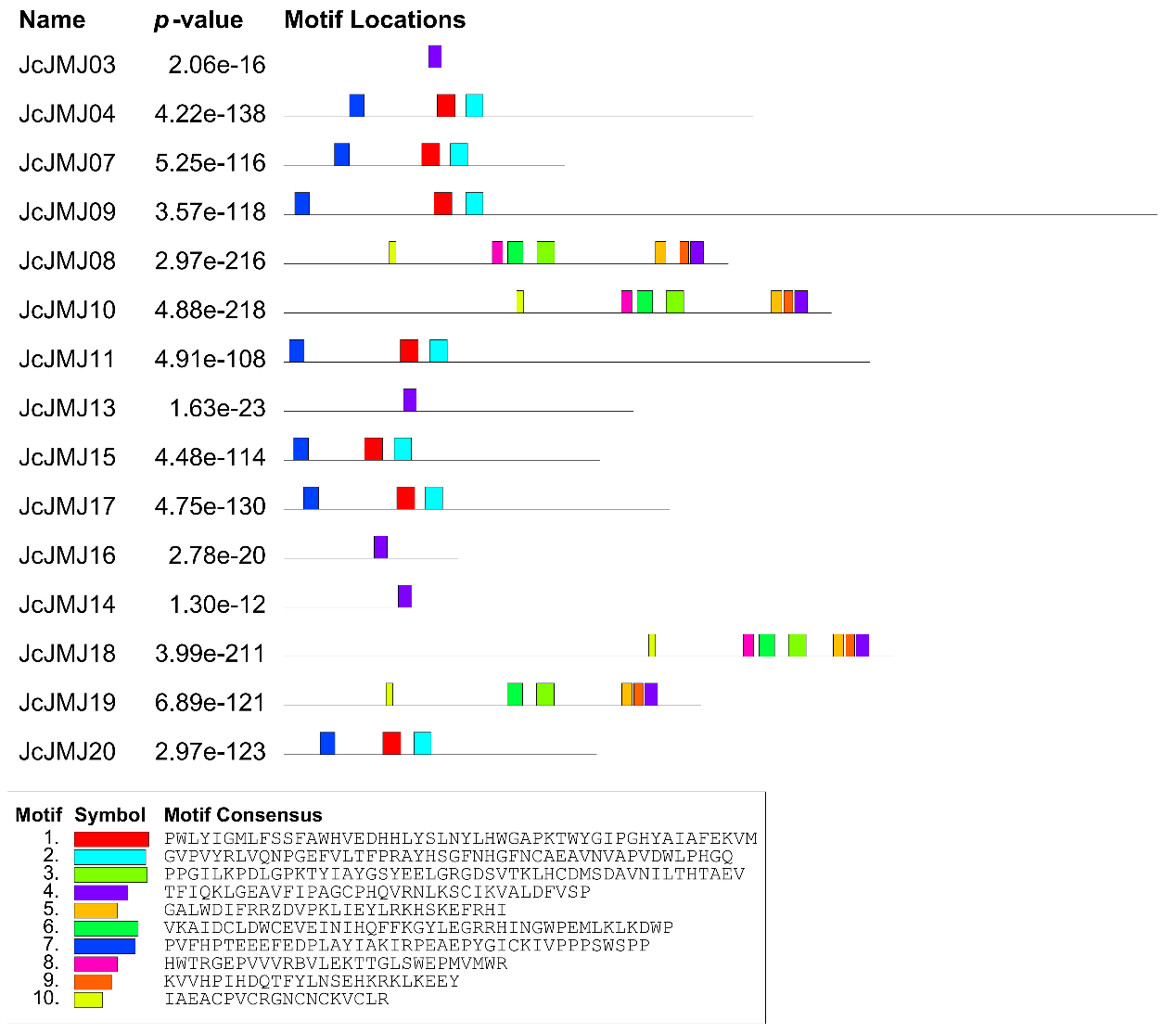


**Figure S2.** The detailed motif symbol and the corresponding motif consensus.

**
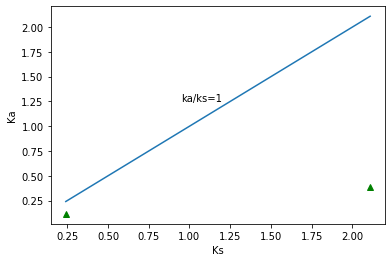
**

**Figure S3.** The Ka/Ks value of duplicated JcJMJ gene pairs. The x and y axes denote the Ks and Ka values for each pair. The Blue line represent Ka/Ks = 1.

**
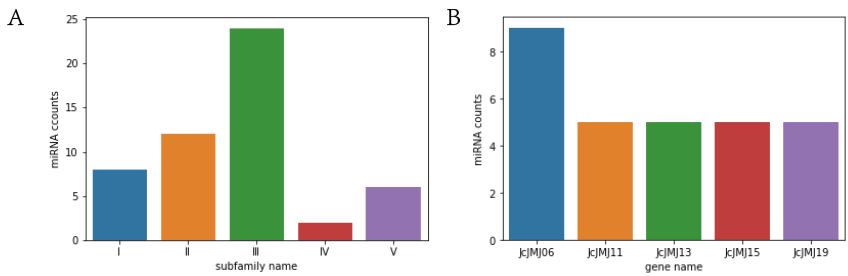
**

**Figure S4.** miRNA counts. (A) shows the five subfamilies regulated by miRNAs, and (B) shows the top five JcJMJ genes with the most miRNA-regulated target sites.
